# Prooxidant-Antioxidant Balance in Umbilical Cord Blood of Infants with Meconium Stained of Amniotic Fluid

**DOI:** 10.1155/2013/270545

**Published:** 2013-11-28

**Authors:** Mohammad Hassan Arjmand, Farhat Ahmad Shah, Masoud Saleh Moghadam, Fatemeh Tara, Amin Jalili, Mojtaba Mosavi Bazaz, Daryoush Hamidi Alamdari

**Affiliations:** ^1^Department of Biology, School of Sciences, Payame Noor University, Mashhad 91735433, Iran; ^2^Neonatal Research Center, Mashhad University of Medical Sciences, Mashhad 9137913316, Iran; ^3^Department of Chemistry, School of Science, Payame Noor University, Mashhad 91735433, Iran; ^4^Department of Obstetrics and Gynecology, OM-Albanin Hospital, Mashhad University of Medical Sciences, Mashhad 9144663595, Iran; ^5^Department of New Science and Technologies, Mashhad University of Medical Sciences, Mashhad 9177948564, Iran; ^6^Department of Social Medicine, Faculty of Medicine, Mashhad University of Medical Sciences, Mashhad 9138913131, Iran; ^7^Stem Cell and Regenerative Medicine Research Group, Department of Biochemistry, Faculty of Medicine, Mashhad University of Medical Sciences, Mashhad 9177948564, Iran

## Abstract

*Objective*. Using a novel assay termed prooxidant-antioxidant balance (PAB) assay to determine prooxidant-antioxidant balance in umbilical cord blood of infants with meconium stained of amniotic fluid (MSAF). Passage of meconium in amniotic fluid is associated with increase of neonatal mortality and morbidity. This complication occurs in about 15% of infants and is more widespread in postterm neonates. About 1.5 percent of neonates with MSAF develop meconium aspiration syndrome. *Method*. Sera of 29 umbilical cord blood of infants with MSAF and 32 healthy infants (HI) were collected. Both groups had nonsmoker and non-alcoholic mothers with no diseases. The PAB was measured. *Result*. There was a significant increase of PAB value (32.8 ± 15.9 HK) in umbilical cord blood of infants with MSAF in comparison to HI (24.5 ± 12.6 HK) (*P* < 0.05). There was no significant correlation between PAB value and age of mothers. *Conclusion*. The increased PAB value in infants with MSAF showed that these infants are exposed to oxidative stress. Further research with larger population is needed to demonstrate the oxidative stress in infants with MSAF.

## 1. Introduction

Meconium is composed of desquamated cells from the intestine and skin, gastrointestinal mucin, lanugo hair, fatty material from the vernix caseosa, amniotic fluid, and intestinal secretions. It also contains blood group-specific glycoproteins, biliary acids (cholic, chenodeoxycholic, deoxycholic, and lithocholic), copper, zinc, magnesium, calcium iron, phosphorus, and plasma proteins such as alpha1-antitrypsin and phospholipase A2. Black-green color of meconium is due to the presence of bile pigments [1, 2]. Expulsion of meconium from the intestinal lumen into the amniotic cavity is a consequence of increased intestinal peristalsis and of anal sphincter relaxation resulting from vagal stimulation [3].

Meconium-stained amniotic fluid (MSAF) can cause mechanical obstruction of airways and pulmonary air leak, pneumonitis, vasoconstriction of pulmonary vessels, and inactivation of surfactant effect which could result in pulmonary inflammation and apoptosis. 7% to 20% of deliveries at term have meconium in the amniotic fluid, which would reach to 40% in postterm deliveries [4]. 5% of infants born through MSAF develop meconium aspiration syndrome (MAS) which is a real threat to many newborns worldwide, with a case fatality rate of 5% (as much as 40%), in addition to MAS short- and long-term pulmonary and neurodevelopmental sequelae which could occur [5, 6].

In human, there are the numerous prooxidants (POX) and antioxidants (AO), and a delicate balance between the production and the elimination of POX is maintained. Oxidative stress (OS) is defined as an imbalance between POX and AO in favor of POX. POX (O_2_^−^, H_2_O_2_, OH^−^, etc.) derive either from metabolic processes or from external sources and can potentially react with the body's own molecules. AO mop up the excess amount of the POX before they damage the essential molecules. AO consists of the soluble antioxidants (vitamin C, urate, etc.), the lipid soluble antioxidants (vitamin E, A, etc.), and the enzymatic antioxidants (catalase, peroxidase, dismutase, etc.) [7].

The concentration of either POX or AO can be measured one by one in separate individual assays. For example, Ochi and Cutler invented a diagnostic plot derived from the measurement of 82 assays which characterize both the oxidative stress and the antioxidant profile [8]. However, the effect of the prooxidant or the antioxidant molecules in serum is an additive effect which could lead to incorrect measurements. Consequently, various methods have been developed in order to measure the total oxidants (such as TOC and TOS assays) or antioxidants (such as FRAP and ORAC assays), separately. In this context, PAB assay is developed to measure the balance of oxidants and antioxidants simultaneously in one experiment and give a redox index [7, 9, 10].

Growing evidence indicates that chronic and acute overproduction of POX under pathophysiologic conditions is unifying mechanism for tissue damage and cell apoptosis. A few studies showed that there is an oxidative stress in MSAF, but more studies using different methods are needed to substantiate the PAB in MSAF.

To the best of our knowledge, this is the first time that the prooxidant-antioxidant balance is measured in umbilical cord blood of infants with MSAF.

## 2. Methods

### 2.1. Chemicals

TMB powder (3,3′,5,5′-Tetramethylbenzidine, Fluka), peroxidase enzyme (Applichem: 230 U/mg, A3791, 0005, Darmstadt, Germany), chloramine T trihydrate (Applichem: A4331, Darmstadt, Germany), hydrogen peroxide (30%) (Merck). Molecular biology grade reagents were used, and preparations were done in double distilled water.

### 2.2. Subjects

Sera of 29 umbilical cord blood of infants with MSAF and 32 healthy infants were collected. Collection of samples was done from July to December in 2013. Both groups had nonsmoker and nonalcoholic mothers without any special diseases. The PAB was measured. The study protocol was approved by the Ethics Committee for Clinical Research of the Mashhad University of Medical Sciences (MUMS).

### 2.3. Collection of Serum

Blood samples from each subject were collected from infant's umbilical cord and then sent to the laboratory for serum separation. Samples were centrifuged at 1500 g for 15 min at room temperature to obtain serum. Hemolytic samples were excluded from analysis. Sera were stored at −80°C.

### 2.4. Method

The PAB assay is the only available test that can measure the balance of oxidants and antioxidants simultaneously in one experiment. It uses two different kinds of reactions: one is an enzymatic reaction where the chromogen TMB is oxidized to a color cation by peroxides and the second is a chemical reaction where the TMB cation is reduced to a colorless compound by antioxidants [7, 8, 11]. The photometric absorbance is then compared with the absorbances given by a series of standard solutions that are made by mixing varying proportions (0–100%) of hydrogen peroxide with uric acid [7]. A low PAB value means that antioxidants are present at greater concentration than oxidants, while a high PAB value means more oxidants are present than antioxidants. The standard solutions were prepared by mixing varying proportions (0–100%) of 250 mM hydrogen peroxide with 3 mM uric acid (in 10 mM NaOH). The TMB powder (60 mg) was dissolved in 10 mL DMSO. For preparation of the TMB cation solution, 400 mL of the TMB/DMSO solution was added to 20 mL of acetate buffer (0.05 M buffer, pH 4.5), and then 70 mL of fresh chloramine T (100 mM) solution was added. The solution was mixed well and incubated for 2 hours at room temperature in a dark place, and then 25 U of peroxidase enzyme solution was added. This mixture was dispensed into 1 mL aliquots and stored at −20°C. The TMB solution was prepared by adding 200 *μ*L TMB/DMSO to 10 mL of acetate buffer (0.05 M buffer, pH 5.8) and the working solution was prepared by mixing 1 mL TMB cation solution with 10 mL TMB solution. This working solution was incubated for 2 min at room temperature in a dark place and immediately used. Ten microliters of each sample, standard, or blank (distilled water) were mixed with 200 *μ*L of working solution in each well of a 96-well plate, which was then incubated in a dark place at 37°C or 12 min. At the end of incubation, 100 *μ*L of 2 N HCl was added to each well, and the optical density (OD) was measured with an ELISA reader at 450 nm, with a reference wavelength of 620 or 570 nm. A standard curve was generated from the values of the standard samples. The values of the PAB assay are expressed in arbitrary units, based on the percentage of hydrogen peroxide in the standard solution. The values of the unknown samples were then calculated based on the values obtained from the generated standard curve.

## 3. Statistics

The Statistical Package for the Social Sciences (SPSS) version 16.0 was used for statistical analysis. All parameters were given as mean ± S.D. The group comparisons were assessed by the independent *t*-test and also Pearson correlation was used for correlation of age and PAB value. The significance level was considered less than 0.05 with a confidence interval of 95%.

## 4. Results

**Figure 1:**
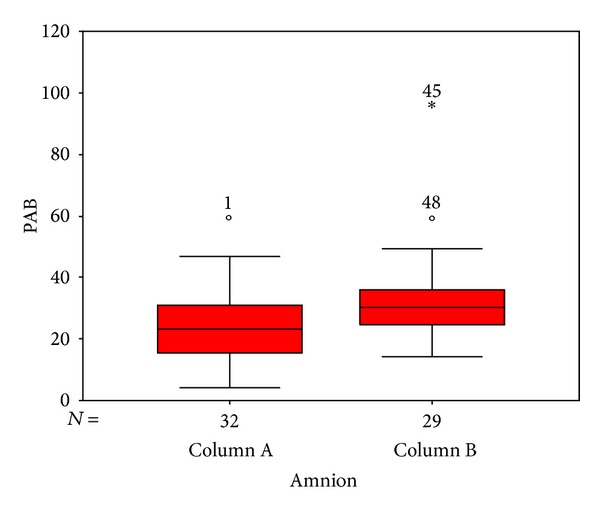
PAB value in HI group (column A) and MSAF group (column B). (Mean and the standard error/standard deviation of PAB value.)

The PAB values of HI group and MSAF group were 24.5 ± 12.6 (HK unit) and 32.8 ± 15.9 (HK unit), respectively (Figure 1). HK is an arbitrary unit used by inventors of PAB method (Hamidi and Koliakos) [7]. There was a significant difference (*P* value = 0.027) between the PAB value of the HI group and the MSAF group. There was no significant correlation between PAB value and the age of mothers (Table 1).

## 5. Discussion

In this study, we showed that there is an increased OS in umbilical cord blood of infants with MSAF in comparison to healthy group using a novel method called PAB assay.

There are two prevailing theories about mechanism of meconium passage in amniotic fluid at term and postterm infants: one is that normal maturation of the gastrointestinal tract results in meconium passage, the other is that pathologic processes such as stress via hypoxia or some infection in fetus can trigger meconium passage. Previous studies have showed an increased OS in hypoxic fetuses and neonates with elevated products of lipid peroxidation in expired air, serum malondialdehyde, serum isoprostanes, serum total hydroperoxides, advanced oxidative protein products, and increased nonprotein bound iron in serum. Low levels of antioxidants have also been observed in red blood cells [12, 13].

Just one study showed that infants with MSAF have high concentration of 8-iso-prostaglandin F2alpha in neonatal cord blood, as a marker of lipid oxidation, and suggests that these infants were exposed to OS [14].

It is demonstrated that there is an increased level of proinflammatory cytokine such as IL-1*β*,  IL-6, tumor necrosis factor (TNF)-*α*, and IL-8 in newborns with MASF [15]. In the other hand, there is a strong correlation between proinflammatory cytokine and OS in various diseases such as cardiovascular disease [16], diabetes mellitus [17], inflammatory bowel disease [18], obesity [19], Alzheimer [20], and alcoholic liver disease [21]. OS can cause increasing cytokine production by many different mechanisms. The increased POX levels, acting similar second messengers, are well known to mediate inflammatory signaling by activating various protein kinases such as JNK, PI3 K, PKC, and PLC. These kinases could stimulate redox sensitive transcription factors such as STAT, CREB, NF-*κ*B, AP-1, NFAT, and ATF2 via a series of signaling events transduced by other kinases like MAPK, ERK, and JAK. The activation of transcription factors leads to the transcriptional activation of inflammatory cytokines (TNF*α*, IL-1, IL-6, IL-8, IL-18, etc.), chemokines (chemoattractant protein-1, etc.), and growth factors (transforming growth factor-*β*, monocyte, connective tissue growth factor, etc.) which could amplify inflammatory complications via autocrine and paracrine pathways [22]. In addition, it is demonstrated that antioxidants can downregulate the proinflammatory cytokines through two possible mechanisms; firstly, through their effect on transcription factors that are regulated by redox status, and secondly by influencing PGE2 synthesis, which plays a key role in Th1 response and regulation of proinflammatory cytokines [23]. Therefore, it is reasonable to conclude that proinflammatory cytokine and OS could interact and provoke each other and contribute to the development and progression of some disease.

Our early results showed that infants with MSAF are exposed with OS. However, larger population is needed to confirm these early results. In addition to that, further research will be necessary to precisely determine the correlation between proinflammatory cytokines and OS in infants with MSAF.

## Figures and Tables

**Table 1:** Age of mothers and PAB value in MSAF and control group.

Group	Ages of mothers (years)*	Number of mothers	PAB value (HK unit)**
MSAF	26 ± 5.6	29	32.8 ± 15.9
Control	25.8 ± 4.4	32	24.5 ± 12.6

*No significant difference between ages of mothers; **significant difference between PAB values (*P* < 0.05).

## Abbreviations

AP1: Activator protein 1
JNK: c-Jun N-terminal kinase
PI3K: Phosphatidylinositol 3-kinase
NF-*κ*B: Nuclear factor-*κ*-binding protein
MAPK: Mitogen-activated protein kinase
PKC: Protein kinase C
PLC: Phospholipase C
ERK: Extracellular signal-regulated kinases
JAK: Janus kinase
STAT: Signal transducer and activator of transcription
NFAT: Nuclear factor of activated T-cells
ATF: Activating transcription factor
PGE2: Prostaglandin E2
IL2: Interleukin-2
CREB: cAMP response element-binding protein
TNF*α*: Tumor necrosis factor alpha
PGE2: Prostaglandin E2
TOC: Total oxidant capacity
TOS: Total oxidant status
FRAP: The ferric reducing ability of plasma
ORAC: Oxygen radical absorbance capacity.
